# Characterization of the complete chloroplast genome of an annual halophyte, *Chenopodium glaucum* (Amaranthaceae)

**DOI:** 10.1080/23802359.2019.1687041

**Published:** 2019-11-08

**Authors:** Yan Yao, Xiao-Tong Li, Xi-Yue Wu, Shou-Jin Fan, Xue-Jie Zhang, Xiao-Jian Qu

**Affiliations:** Key Lab of Plant Stress Research, College of Life Sciences, Shandong Normal University, Ji’nan, Shandong, China

**Keywords:** *Chenopodium glaucum*, plastome, phylogenomics

## Abstract

The complete chloroplast genome (plastome) of *Chenopodium glaucum*, an annual halophytic herb, was determined. The plastome was 152,191 bp in size, containing a large single-copy region (83,675 bp), a small single-copy region (18,130 bp), and two inverted repeats regions (25,193 bp). The overall GC content of this plastome was 37.2%. In total, 113 unique genes were annotated including 79 protein-coding genes (PCGs), 30 tRNAs and 4 rRNAs. Phylogenomic analysis showed that *C. glaucum* was sister to *C. album*.

*Chenopodium glaucum* is an annual halophytic herb from Amaranthaceae with worldwide distribution. Halophytes such as *Suaeda salsa* has succulent leaf to adapt to saline conditions, while *C. glaucum* has inconspicuous morphological specialization under salt stress (Li et al. 2008; Sui et al. 2010; Yang et al. 2010; Song et al. 2011; Li et al. 2012; Cheng et al. 2014; Guo et al. 2015; Song and Wang 2015; Sui 2015; Wang et al. 2015; Chen et al. 2016; Song et al. 2016; Zhou et al. 2016; Song et al. 2017; Sui et al. 2017; Guo et al. 2018; Liu et al. 2018). In addition, *C. glaucum* also has positive environmental impacts such as helping to improve the soil texture and reduce soil salinity, and its leaves can be used as feed (Hong et al. 2017). In this study, we reported the plastome of *C. glaucum*, which would provide fundamental genetic resources for studying this important species as well as resolving its phylogenetic position.

Fresh leaves of *C. glaucum* were collected from Laishan District (Shandong, China; 121°23′N, 37°22′E). Voucher specimen (330138) was deposited at Kunming Institute of Botany, Chinese Academy of Sciences. The modified CTAB method was used to extract total genomic DNA (Wang et al. 2013). Considering the limited fresh sample, chloroplast DNA was not extracted directly (Liu et al. 2017). Total genomic DNA was used for library preparation and paired-end (PE) sequencing by the Illumina MiSeq at Novogene (Beijing, China). Plastome was assembled using Organelle Genome Assembler (OGA; Qu 2019). Annotation was performed with Plastid Genome Annotator (PGA; Qu et al. 2019), coupled with manual correction using Geneious v8.0.2. A maximum-likelihood (ML) tree was reconstructed to determine the phylogenetic placement of *C. glaucum* using RAxML v8.2.10 (Stamatakis 2014), including tree robustness assessment using 1000 rapid bootstrap replicates with the GTRGAMMA substitution model, based on alignment of 79 shared protein-coding genes using MAFFT v7.313 (Katoh and Standley 2013).

The complete plastome of *C. glaucum* (GenBank accession number: MN422308) was 152,191 bp in length, composed of a large single-copy region (83,675 bp), a small single-copy region (18,130 bp), and a pair of inverted repeats (25,193 bp). The overall GC content was 37.2%. A total of 113 unique genes were annotated in this plastome, including 79 PCGs, 30 tRNAs, and 4 rRNAs. Thirteen PCGs and eight tRNAs contained introns among the annotated genes. The ML phylogenetic tree showed that *C. glaucum* was sister to *C. album* (Figure 1).

**Figure 1.**
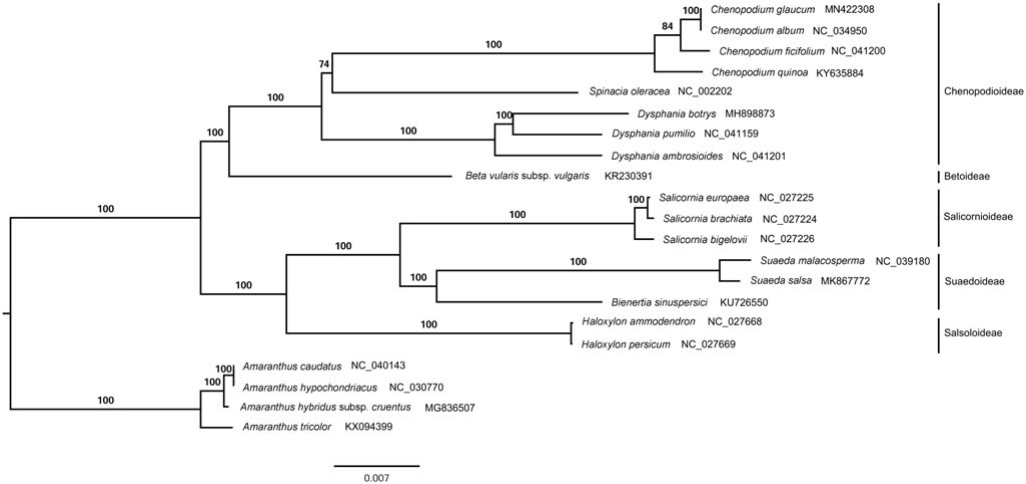
A maximum-likelihood (ML) tree inferred from 79 plastome genes. Four *Amaranthus* species from Amaranthaceae are used as outgroup. The numbers on branches are bootstrap support values.
